# Role of selenium addition to CdZnTe matrix for room-temperature radiation detector applications

**DOI:** 10.1038/s41598-018-38188-w

**Published:** 2019-02-07

**Authors:** U. N. Roy, G. S. Camarda, Y. Cui, R. Gul, A. Hossain, G. Yang, J. Zazvorka, V. Dedic, J. Franc, R. B. James

**Affiliations:** 1 Brookhaven National Laboratory, Upton, NY 11973 USA; 2 Institute of Physics, Charles University, Ke Karlovu 5, Prague, 121 16 Czech Republic; 3 Present Address: North Carolina State University, Raleigh, NC 27695-7909 USA; 4 Present Address: Savannah River National Laboratory, Aiken, SC 29808 USA

## Abstract

Because of its ideal band gap, high density and high electron mobility-lifetime product, cadmium zinc telluride (CdZnTe or CZT) is currently the best room-temperature compound-semiconductor X- and gamma-ray detector material. However, because of its innate poor thermo-physical properties and above unity segregation coefficient for Zn, the wide spread deployment of this material in large-volume CZT detectors is still limited by the high production cost. The underlying reason for the low yield of high-quality material is that CZT suffers from three major detrimental defects: compositional inhomogeneity, high concentrations of dislocation walls/sub-grain boundary networks and high concentrations of Te inclusions/precipitates. To mitigate all these disadvantages, we report for the first time the effects of the addition of selenium to the CZT matrix. The addition of Se was found to be very effective in arresting the formation of sub-grain boundaries and its networks, significantly reducing Zn segregation, improving compositional homogeneity and resulting in much lower concentrations of Te inclusions/precipitates. Growth of the new quaternary crystal Cd_1−x_Zn_x_Te_1−y_Se_y_ (CZTS) by the Traveling Heater Method (THM) is reported in this paper. We have demonstrated the production of much higher yield according to its compositional homogeneity, with substantially lower sub-grain boundaries and their network, and a lower concentration of Te inclusions/precipitates.

## Introduction

X-ray and gamma-ray detectors have broad applications, ranging from medical imaging, non-proliferation and national security to astrophysics. Because of the favorable physical properties and excellent optoelectronic properties, CZT and CdTe are the leading room-temperature radiation detector materials offering promising energy resolution^1–7^. High-quality CZT materials with large volume, low defects and compositional uniformity are needed for such applications, and their availability continues to be a major challenge for high-yield production. Despite recent significant improvements in the quality of CZT materials, the CZT technology still suffers from three major detrimental defects, i.e., high concentrations of sub-grain boundaries and their networks, Te inclusions/precipitates and compositional inhomogeneity^1,8–14^, which are the key issues for realization of large-volume detectors at a lower production cost. Poor thermo-physical properties of the melt and the solidified material near or below the melting point of CdTe/CZT are responsible for the generation of high concentrations of sub-grain boundaries and their networks during growth and post-growth ingot cooling processes. The sub-grain boundaries distribute randomly in the CZT/CdTe matrix and severely affect the charge collection as they act as charge trapping centers^10,15–17^. Consequently, these defects adversely affect the charge-collection efficiency and energy resolution of the devices^10,18^. Due to these cumulative effects, the performance degradations of the devices are more pronounced for thick detectors. The Te inclusions/precipitates embedded in the CZT/CdTe matrix, generally known as secondary phases, also act as trapping centers and are responsible for severe degradation of the spectroscopic performance of the devices, particularly for long-drift length (thicker) devices^19,20^. The mobility-lifetime product (µτ) values of electrons were found to vary by a factor of ten between clean regions and regions with relatively high Te inclusions^19^. Such spatial non-uniformity of the charge-collection efficiency is responsible for variability in the pulse-height spectra, depending on the drift paths of the ionized electrons, resulting in broadened photopeaks. Both experimental and simulation results showed the severe performance degradation of the device performance due to the presence of Te inclusions of different size distribution and concentrations^21^. It is to be noted that the sub-grain boundaries were also found to be decorated with the Te inclusions/precipitates^10,22^. Though the Te inclusions can be eliminated by annealing under cadmium vapor, the process produces star-like defects that are not visible in infra-red (IR) transmission microscopy. These star-like defects are 50–100 times larger than the Te inclusions and also act as trapping centers, which can severely affect the charge-transport properties^23^.

To mitigate these problems presently suffered by CZT material, we adopted the growth of a new quaternary material Cd_1−x_Zn_x_Te_1−y_Se_y_ (CZTS). The addition of selenium was found to be very effective in suppressing the formation of sub-grain boundaries and their networks, reducing the concentration of Te inclusions and improving compositional homogeneity along the length of the grown ingots.

## Results

**Figure 1:**
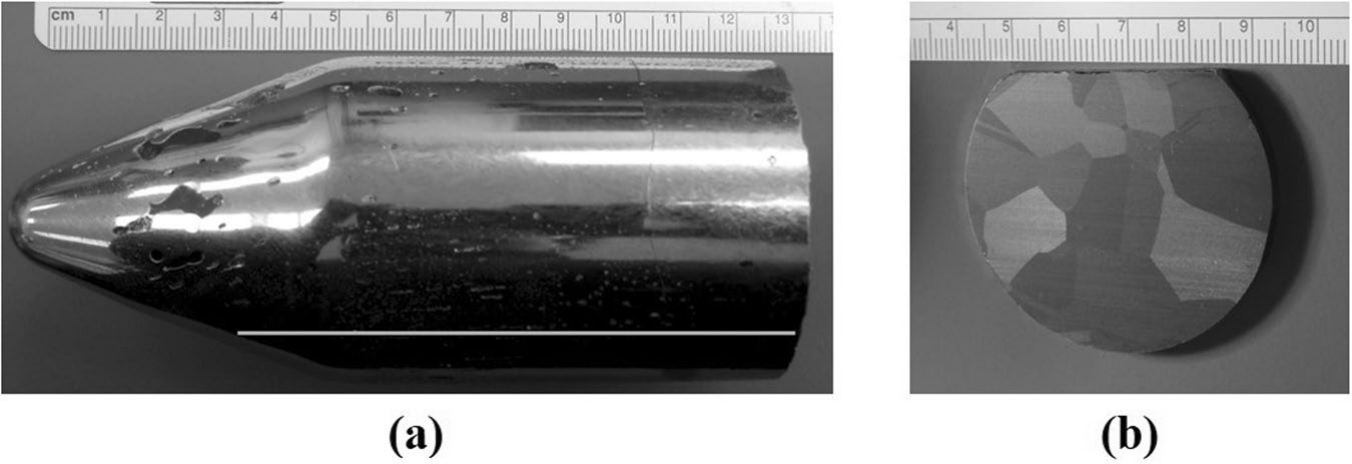
Photograph of the (**a**) THM grown two-inch diameter Cd_0.9_Zn_0.1_Te_0.93_Se_0.07_ ingot and (**b**) a cross-sectional slice of the ingot.

We have grown two-inch diameter Cd_0.9_Zn_0.1_Te_0.93_Se_0.07_ ingots by the Traveling Heater method (THM). Figure 1a shows the photograph of a Cd_0.9_Zn_0.1_Te_0.93_Se_0.07_ ingot grown by the THM.

**Figure 2:**
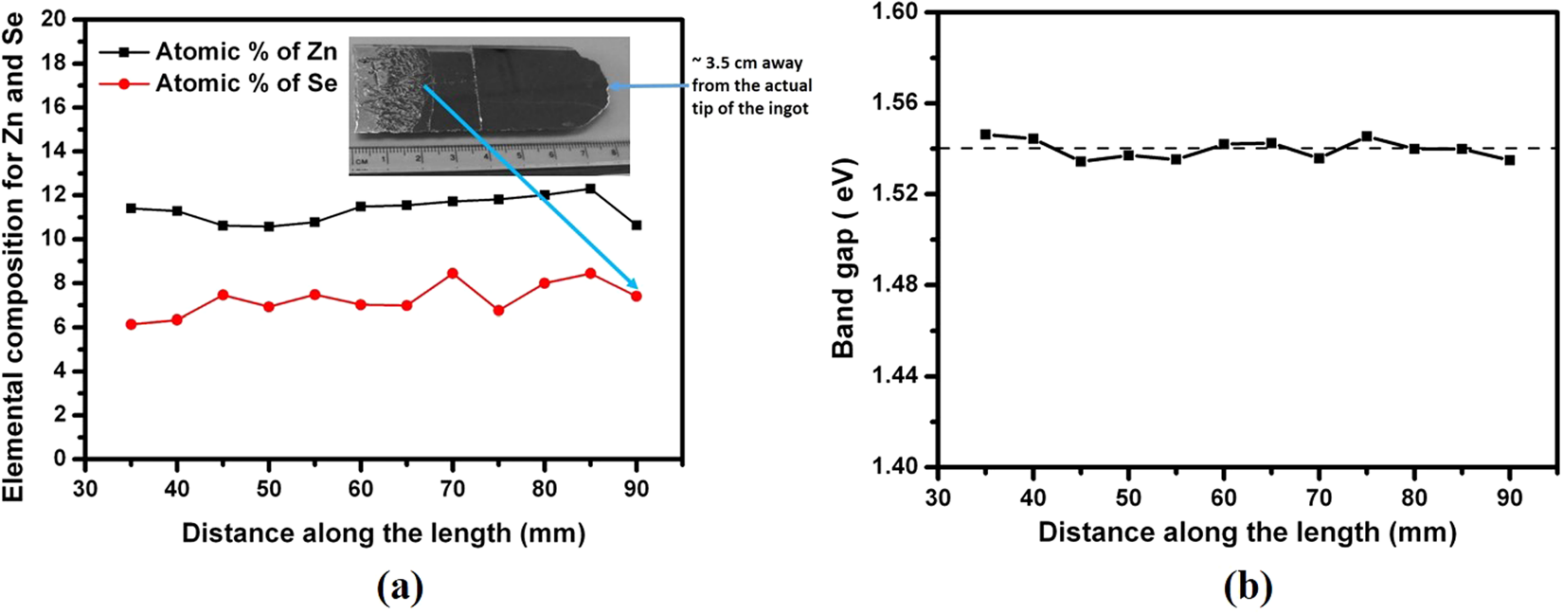
(**a**) Axial Se and Zn composition (Atomic %) of the as-grown Cd_0.9_Zn_0.1_Te_0.93_Se_0.07_ ingot (inset shows the sample cut along the length of the ingot), and (**b**) calculated band-gap along the length of the ingot.

The ingot weighs about 1 kg and has the dimensions of 52 mm in diameter and 11 cm in length. The ingot was polycrystalline in nature with large grains, and the cross-sectional picture of the as-cut wafer sliced perpendicular to the growth direction is shown in Fig. 1b. The composition analyses of the ingot along the length, were performed on the wafer cut along the length of the ingot as indicated by the white line in Fig. 1a. Considering the accuracy of Energy Dispersive X-ray Analysis (EDAX) to be about ±1 atomic %^24^, the composition is fairly constant throughout the length of the ingot as shown in Fig. 2a. The optical photograph of a sample cut along the length of the ingot is shown in the inset of Fig. 2a. A slight dip in concentration of Zn and Se was observed near the vicinity of the Te + CZTS/CZTS interface as indicated by the blue arrow in Fig. 2a, while the concentration of Zn was found to decrease drastically from near the middle of the ingot towards the interface in CdZnTe ingot grown by similar THM technique^12^. It is thus likely that selenium plays a role in modifying the segregation of Zn in the CZT matrix offering high compositional homogeneity along the length of CZTS ingot, compared to CZT, in which only about one third of the ingot usually has the desired uniformity in the Zn concentration. Consequently, since the zinc concentration is more homogeneous over the entire length of the CZTS ingot, the yield of CZTS is expected to be much higher than in CZT ingots grown by the THM technique. From the measured composition, the band-gap of the material was estimated along the length of the ingot using the empirical formula^25^ for the Cd_1−x_Zn_x_Te_1−y_Se_y_ quaternary compound.

1 $${{\rm{Eg}}}_{({\rm{x}},{\rm{y}})}=1.511-0.54{\rm{y}}+0.6{\rm{x}}({\rm{x}},{\rm{y}}\le 0.10)$$

**Figure 3:**
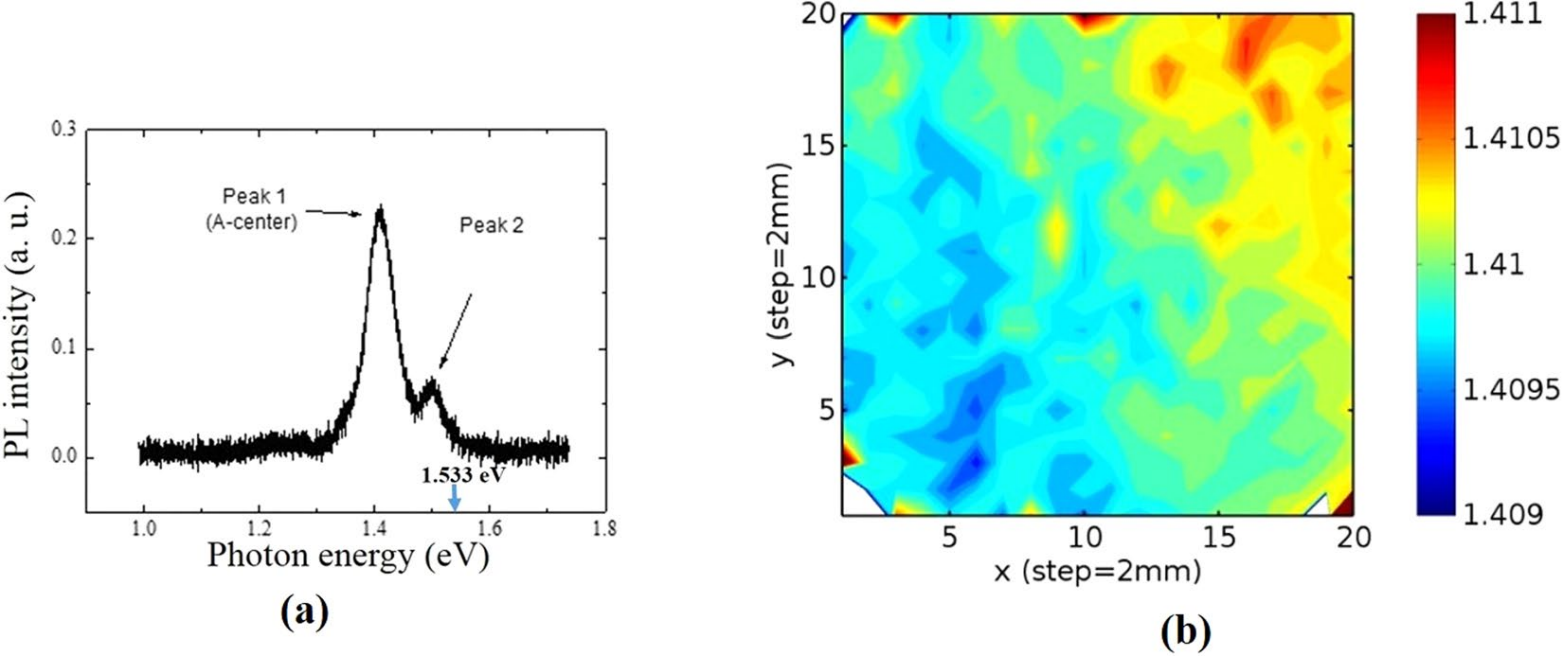
(**a**) Typical PL spectrum at room temperature and, (**b**) mapping of peak energy positions (of peak 1) over 4 × 4 cm^2^ area of the two-inch Cd_0.9_Zn_0.1_Te_0.93_Se_0.07_ wafer grown by THM.

Considering the experimental accuracy of EDAX measurements, the calculated band-gap derived from the EDAX composition measurements is fairly uniform along the entire length of the CZTS ingot as shown in Fig. 2b. The compositional homogeneity of the cross-sectional wafer of the two-inch diameter CZTS ingot was also studied by room-temperature photoluminescence (PL) mapping. The peak energy position of the PL spectrum is very sensitive to the band-gap of the material, and hence the compositional variation. A typical room-temperature PL spectrum is shown in Fig. 3a. The calculated band-gap of the quaternary compound Cd_0.9_Zn_0.1_Te_0.93_Se_0.07_ is estimated as 1.533 eV by equation 1 and shown by the blue arrow. The peak 1 centered at ~1.41 eV is ~0.123 eV above the valence band edge as indicated in Fig. 3a, is dominant and is assigned as the A-center, which is the Cd vacancy-In complex. The absence of the near band edge luminescence in the spectrum is expected, as the near band edge peaks are known to diminish near room temperature due to thermal quenching^26^ for CZT. The A-center in Cd_0.9_Zn_0.1_Te is located (0.12–0.15) eV above the valence band edge, and for CdTe the corresponding position of the A-center is ~(0.11–0.14) eV^27^. The origin of the peak 2 is presently unknown, but might be due to the donor-acceptor pair (DAP) transition. The mapping of the peak energy position (peak 1) after Gaussian fitting was carried out over the area of 4 × 4 cm^2^ in the two-inch wafer. The mapping of the energy position of peak 1 over a 16-cm^2^ area in the Cd_0.9_Zn_0.1_Te_0.93_Se_0.07_ wafer is shown in Fig. 3b. As is evident, the peak energy position varies between 1.409 eV to 1.411 eV, and the absolute variation of the peak energy is ∆E = 2 meV over the entire area. This suggests very high compositional uniformity in the material. As opposed to EDAX, the sensitivity/accuracy of the PL peak-position mapping is very high, and our PL results depict very high compositional uniformity across the diameter of the ingot. It is to be noted that, if there is any compositional variation along the length of the ingot, it reflects radial compositional variation as well unless the growth interface is perfectly flat^11^. Thus, the very high radial compositional homogeneity also depicts the same along the length of the ingot, as our growth interface is highly concave as shown in the inset of Fig. 2a.

**Figure 4:**
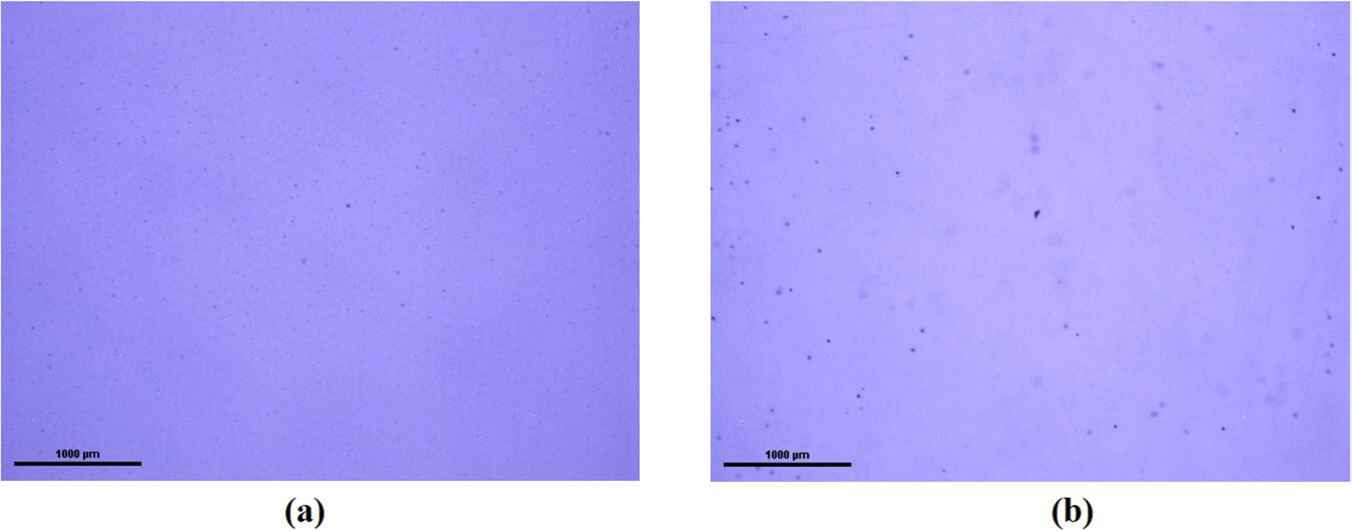
Typical IR transmission microscopic image showing Te inclusions/precipitates, (**a**) and (**b**) different positions.

**Figure 5:**
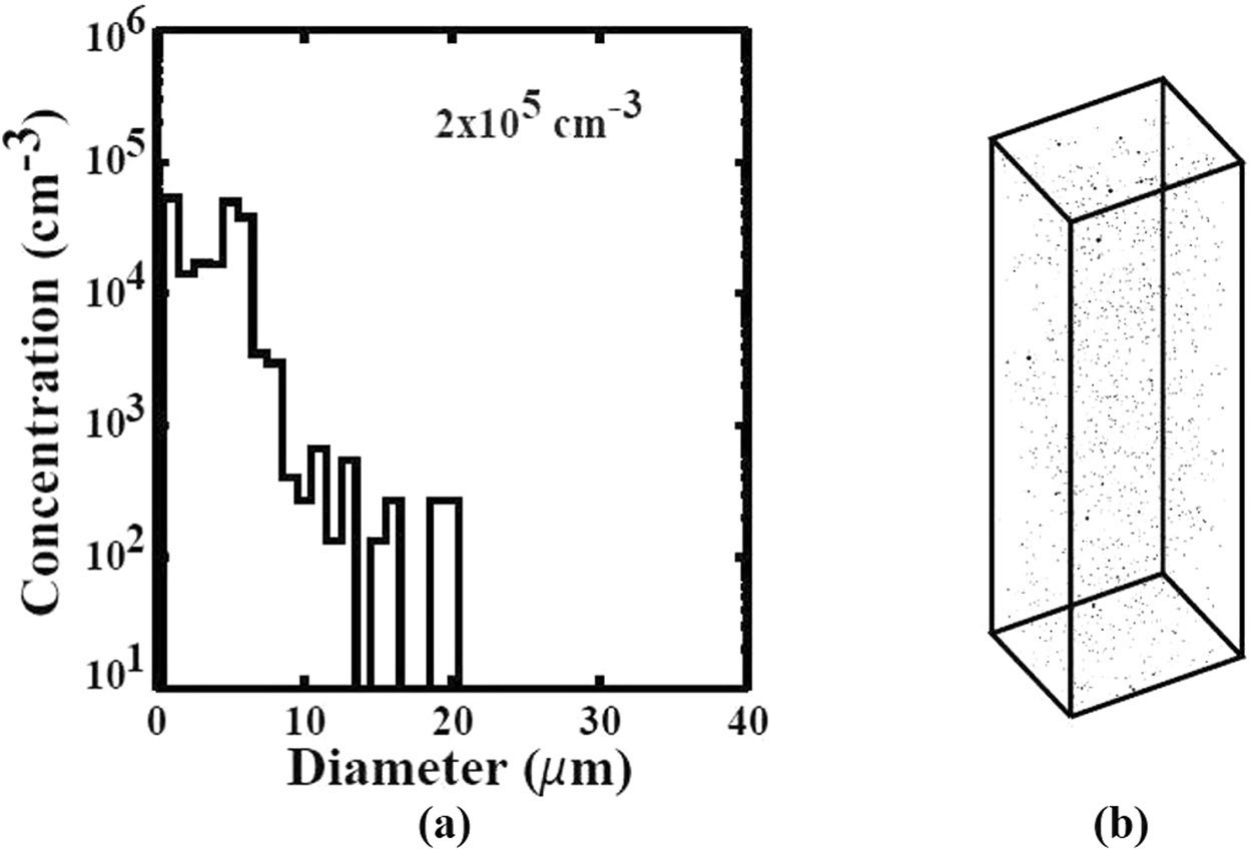
(**a**) Size distribution and concentrations of Te inclusions/precipitates and (**b**) the corresponding 3D distribution for as-grown Cd_0.9_Zn_0.1_Te_0.93_Se_0.07_ sample by THM.

**Figure 6:**
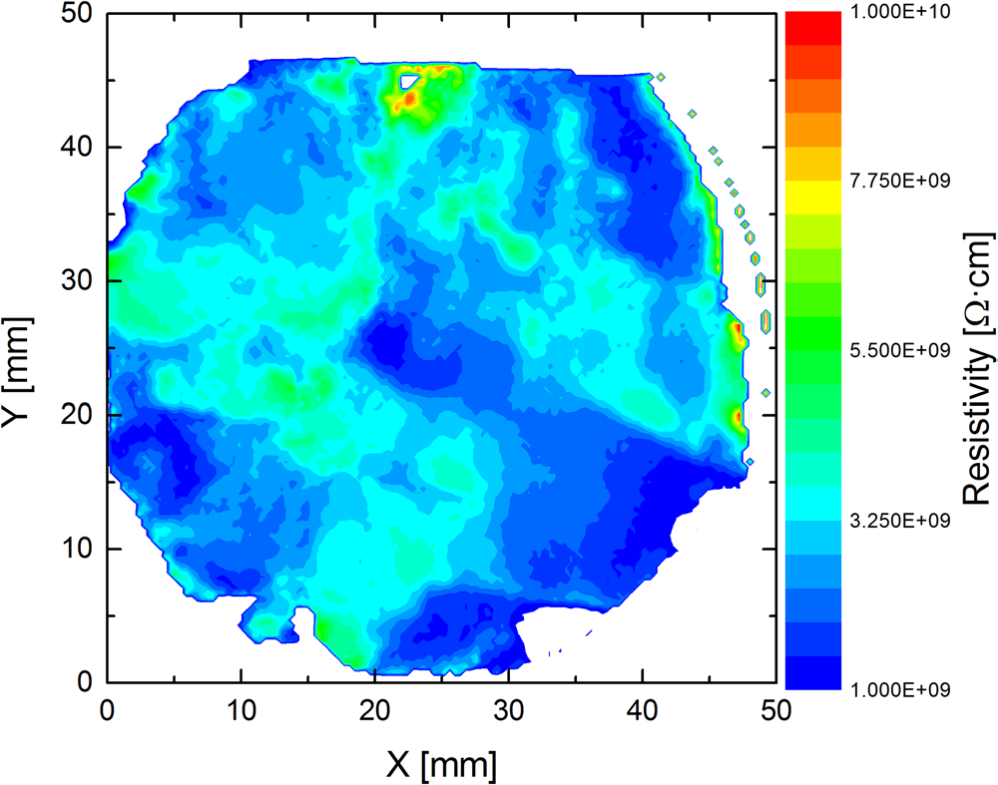
Resistivity map of two-inch diameter THM grown Cd_0.9_Zn_0.1_Te_0.93_Se_0.07_ wafer.

**Figure 7:**
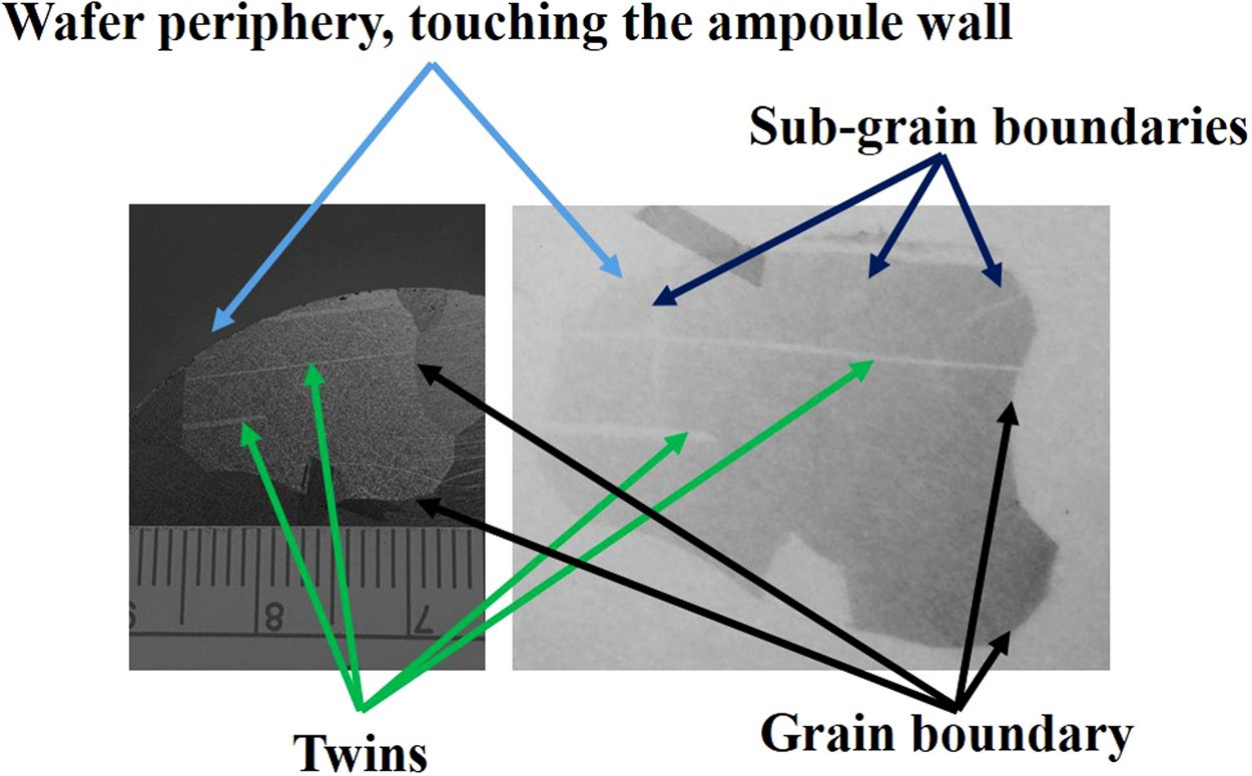
Optical photograph of the grain (left) and the corresponding X-ray topographic image of the grain.

We evaluated the size distribution and concentrations of Te inclusions/precipitates of the THM-grown Cd_0.9_Zn_0.1_Te_0.93_Se_0.07_ samples. Figure 4a and b show typical infra-red (IR) transmission image of the as-grown Cd_0.9_Zn_0.1_Te_0.93_Se_0.07_ sample, taken at two different positions. The Te inclusions/precipitates are visible as the black dots as shown in Fig. 4. The size distribution and concentrations of Te inclusions/precipitates are shown in Fig. 5a. The total concentration was found to be ~2 × 10^5^ cm^−3^, and the concentrations were measured over the volume of ~1.1 × 1.5 × 5 mm^3^, where 5 mm is the thickness of the sample. As evident from Fig. 5a, the concentrations of large Te inclusions in range of ~15–20 µm are ~ 3 × 10^2^ cm^−3^, while the concentration of smaller size inclusions in range of ~1–7 µm is ~8 × 10^4^ cm^−3^. The total average concentrations of Te inclusions/precipitates at five different positions of the sample were found to be ~2.5 × 10^5^ cm^−3^, which is roughly one order of magnitude less than as-grown fast-cooled CZT samples grown by similar technique^28^ (Please see Figs 6 and 7 on this link for comparison, 10.1016/j.jcrysgro.2011.07.025). Figure 5b shows the corresponding 3D distributions of Te inclusions/precipitates over the volume of ~1.1 × 1.5 × 5 mm^3^ for an as-grown Cd_0.9_Zn_0.1_Te_0.93_Se_0.07_ sample.

The resistivity map of 52-mm diameter wafer cut perpendicular to the ingot axis was carried out using a Contactless Resistivity Mapping (CoReMa) technique; the results are shown in Fig. 6. The variation of the resistivity, over the entire area of the wafer was found to be in the range of ~1.5–6 × 10^9^ ohm-cm, which is consistent with high-quality CZT material^13^. However, the absolute resistivity is lower than the desired resistivity (1–3 × 10^10^ ohm-cm) value for most detector applications. Efforts are underway to increase the resistivity of the Cd_0.9_Zn_0.1_Te_0.93_Se_0.07_ material. The sub-grain boundaries and their network were investigated by X-ray topographic analyses of the as-grown Cd_0.9_Zn_0.1_Te_0.93_Se_0.07_ ingot. The X-ray topographic experiments were carried out using a synchrotron beam line at the Advanced Light Source (ALS), Lawrence Berkeley National Laboratory. The X-ray topographic experiments were carried out on samples that were highly polished followed by etching in a 2% bromine-methanol (BM) solution for two minutes. The mechanical polishing might cause a damaged layer below the polished surface, and the damaged layer could result in artifacts and mask many defect features in the topographic image. Thus, etching was carried out to remove any damaged layer that might be produced during polishing. Figure 7 shows the optical photograph of a grain (left) from a two-inch Cd_0.9_Zn_0.1_Te_0.93_Se_0.07_ wafer and the corresponding X-ray topographic image (right) of the same grain. Only a few sub-grain boundaries are evident from the X-ray topographic image, and unlike CZT, (Please see Fig. 11 of X-ray topographic images of CZT from seven different vendors for comparison, and their effect on detector response please see Figs 14 and 15. 10.1016/j.jcrysgro.2013.01.048) no sub-grain boundary network was observed in our Cd_0.9_Zn_0.1_Te_0.93_Se_0.07_ sample. It is to be noted that, even though the ingot was cooled rapidly to room temperature after the termination of growth, no thermal stresses and sub-grain boundary networks are evident in the X-ray topographic image.

This type of image indicates that the selenium plays a major role in effective solution hardening of the CZTS matrix resulting in growth of CZTS free from sub-grain boundary networks. A similar result was also observed for CdTe_x_Se_1−x_ (CTS) ingots grown by the THM technique^29^. It is to be noted that no lattice distortion was observed near the periphery of the wafer touching the ampoule wall, as indicated by the blue arrow, depicting stress-free ampoule wall contact. In general, ampoule walls are the common source of introducing strains^30,31^, and severe lattice distortions near the periphery of the ingots have been observed for CdTe-based materials^30,31^, even for contactless vapor grown CdZnTe^32^. The material is free from any thermal stress as evident from the undistorted twins in the X-ray topographic image as indicated by the green arrows in Fig. 7.

The present experimental results clearly demonstrate the role of Se as a promising additive in CZT matrix for effectively enhancing the compositional homogeneity of the as-grown ingot and reducing the concentration of size and distribution of Te inclusions/precipitations. Selenium was also found to be very effective as a solid solution hardening agent that helps to produce ingots free from sub-grain boundary networks, even for quenched ingots. Better compositional homogeneity, a lower concentration of secondary phases and the absence of sub-grain boundary network in CZTS indicate tremendous potential of CZTS for better detector quality and a much lower production cost.

## Methods

The indium-doped Cd_1−x_Zn_x_Te_1−y_Se_y_ crystals were grown by THM using Te as solvent, with nominal concentration of 10% Zn and 7% Se. The composition (Cd_0.9_Zn_0.1_Te_0.93_Se_0.07_) was synthesized first from using the required proportions of 6 N purity Cd_0.9_Zn_0.1_Te with 10% Zn and 6 N purity CdSe as starting materials. The THM growth was carried out in a two-inch diameter (52 mm ID) quartz ampoule. Prior to loading the required material, the ampoule was coated with carbon by cracking spectroscopic-grade acetone at about 900 °C. The required amount of pre-synthesized Cd_0.9_Zn_0.1_Te_0.93_Se_0.07_ with 6 N purity Te was then loaded in the freshly coated ampoule and sealed under the dynamic vacuum of ~2 × 10^−6^ torr. The growth was carried out in an independently controlled three-zone vertical furnace at a growth rate of ~3 mm/day. The growth was performed at ~850 °C near the growth interface. After completion of the growth, the furnace was switched off to cool down the grown ingot naturally to room temperature.

Macroscopic compositional analyses along the length of the ingot were explored by Energy Dispersive Spectroscopy (EDS) using a Jeol 7600 electron microscope. The PL mapping for the wafer was carried out at room temperature with the exciting wavelength of 1.94 eV using a Radius (Coherent) laser. The sample was studied with an X-Y scanner set at a 2-mm step size. The concentration and size distribution of Te inclusions/precipitates inside the bulk of the as-grown sample were evaluated by IR transmission microscopic images at different depths of the polished wafer; the results were detailed elsewhere^33^. White Beam X-ray Diffraction Topography (WBXDT) analyses were carried out at the Lawrence Berkeley National Laboratory’s Advanced Light Source (LBNL’s ALS) Beamline 3.3.2, with the beam energy ranging from 4 keV to 25 keV. All the topographic analyses were performed on a surface that was etched in 2% bromine-methanol for two minutes to remove any residual damage associated with polishing to a mirror finish.

It is worth mentioning that some findings from the presented studies were presented earlier at SPIE conference in Nov. 2016, San Diego, CA., (10.1117/12.2240430).
